# Book Reviews

**Published:** 1799-12

**Authors:** 


### MISCELLANEOUS.

*Schwedische Annalen,* &c.—The Swedish Annals of Medicine and Natural History. By Dr. RUDOLPHI, &c.

[Concluded from p. 399 of our last Number.]

Art. VI.—X. A review of SVEN HEDIN's Scientific Essays, addressed to physicians aud surgeons. XI. and XII. Of SVEN A. HEDIN's Manual of the practice of Medicine;—a very just and profound specimen of criticism. XIII. A. I. SEGERSTEDT's Elements of Medicine. XIV. B. BJORNLUND *materia medica selecta.* XVII. ADOLPHI MURRAY & NICOL. A. BERGSTEN *usus modioli in fractura et depressione cranii, casu singulari illustratus.*—Among the XLI. articles which this work contains, we shall take notice only of the XVII. entitled, CAR. P. THUNBERG *et* CAR. JO. KJELLMANN, *Diss. de usu menyanthidis trifoliatæ (Trifolii fibrini);* the leaves of which are frequently used by brewers as a substitute for the hop. It is asserted, that the beer in which this plant has been boiled much resembles London Porter.

*Magazin*

*Magazin für gemeinnützige Arzneykunde, &c.*—The Magazine devoted to medical ſubjects of general utility, and medical police. Edited by J. H. Rahn, Member of the Helvetic Republican Senate, No. I. 11½ ſheets 8vo. 1799 (12 gr. or 2s.) *Zürich.* Orell, Fuſsli and Co.

With this work the venerable Editor recommences his periodical labours, which had been interrupted by the late calamitous events in his native country. As our praiſe can add but little to his eſtabliſhed reputation, we ſhall extract merely ſome of the ſubjects treated of in this popular Magazine: I. Propoſals and a Plan of Medical Police-laws for the Helvetic Republic one and indiviſible. III. Inſtructions for Midwives, Fathers, and Mothers; with uſeful practical advice and cautions againſt the prejudices and hurtful cuſtoms prevailing in midwifery; being a review of a popular book, publiſhed at Erlang, in 1798. VII. Tissot's Directions for treating perſons bitten by a mad dog. IX. An Account of ſome epidemic diſeaſes, particularly the malignant ſmall-pox, which prevailed in ſeveral Cantons of the Republic, during the year 1798.

